# Bacterial Selection during the Formation of Early-Stage Aerobic Granules in Wastewater Treatment Systems Operated Under Wash-Out Dynamics

**DOI:** 10.3389/fmicb.2012.00332

**Published:** 2012-09-13

**Authors:** David G. Weissbrodt, Samuel Lochmatter, Sirous Ebrahimi, Pierre Rossi, Julien Maillard, Christof Holliger

**Affiliations:** ^1^Laboratory for Environmental Biotechnology, School for Architecture, Civil and Environmental Engineering, Ecole Polytechnique Fédérale de LausanneLausanne, Switzerland; ^2^Central Environmental Molecular Biology Laboratory, School for Architecture, Civil and Environmental Engineering, Ecole Polytechnique Fédérale de LausanneLausanne, Switzerland

**Keywords:** biological wastewater treatment, aerobic granular sludge, granule formation, wash-out dynamics, bacterial selection, nutrient removal limitations

## Abstract

Aerobic granular sludge is attractive for high-rate biological wastewater treatment. Biomass wash-out conditions stimulate the formation of aerobic granules. Deteriorated performances in biomass settling and nutrient removal during start-up have however often been reported. The effect of wash-out dynamics was investigated on bacterial selection, biomass settling behavior, and metabolic activities during the formation of early-stage granules from activated sludge of two wastewater treatment plants (WWTP) over start-up periods of maximum 60 days. Five bubble-column sequencing batch reactors were operated with feast-famine regimes consisting of rapid pulse or slow anaerobic feeding followed by aerobic starvation. Slow-settling fluffy granules were formed when an insufficient superficial air velocity (SAV; 1.8 cm s^−1^) was applied, when the inoculation sludge was taken from a WWTP removing organic matter only, or when reactors were operated at 30°C. Fast-settling dense granules were obtained with 4.0 cm s^−1^ SAV, or when the inoculation sludge was taken from a WWTP removing all nutrients biologically. However, only carbon was aerobically removed during start-up. Fluffy granules and dense granules were displaying distinct predominant phylotypes, namely filamentous *Burkholderiales* affiliates and *Zoogloea* relatives, respectively. The latter were predominant in dense granules independently from the feeding regime. A combination of insufficient solid retention time and of leakage of acetate into the aeration phase during intensive biomass wash-out was the cause for the proliferation of *Zoogloea* spp. in dense granules, and for the deterioration of BNR performances. It is however not certain that *Zoogloea*-like organisms are essential in granule formation. Optimal operation conditions should be elucidated for maintaining a balance between organisms with granulation propensity and nutrient removing organisms in order to form granules with BNR activities in short start-up periods.

## Introduction

Aerobic granular sludge (AGS) wastewater treatment processes are attractive for intensive and high-rate biological nutrient removal (BNR) and secondary clarification in single sequencing batch reactors (SBR; de Bruin et al., 2004; Giesen et al., 2012). Stable dense and fast-settling aerobic granules with tailored metabolic activities for the removal of carbon, nitrogen, and phosphorus are desired for the operation of robust AGS wastewater treatment plants (WWTP). For instance, the overgrowth of filamentous organisms must be avoided in order to prevent process disturbances by the deterioration of the settling properties of aerobic granules (van Loosdrecht et al., 2008).

The formation of aerobic granules has been stimulated by reactor start-up conditions leading to the wash-out of flocculent biomass and selecting for a fast-settling biomass, namely with the combination of short settling times of 3–5 min and short hydraulic retention times (HRT) of 6 h (Beun et al., 1999). Granulation can be impacted by additional operation parameters such as the influent feeding regime, the hydrodynamic shear force, and the concentration of dissolved oxygen (DO). For a review, refer to Lee et al. (2010). Granules have been successfully cultivated with feast-famine regimes involving pulse feeding (3–5 min) followed by prolonged aeration (3–4 h; Morgenroth et al., 1997; Beun et al., 1999; Tay et al., 2002), or anaerobic feeding (1 h) followed by aerobic starvation (2 h; de Kreuk et al., 2005). Granulation has only been observed with up-flow superficial air velocities (SAV) above 0.010 m s^−1^, typically between 0.025 and 0.045 m s^−1^. High up-flow aeration induce high shear and compaction forces at the surface of granules (Zima et al., 2007), and stimulate the production of exopolymeric substances (EPS) as well as hydrophobic adhesive interactions (Liu and Tay, 2002; Dulekgurgen et al., 2008).

Several studies have however reported on the deterioration of the settling properties of aerobic granules by overgrowth of filamentous microbial structures, called filamentous bulking. For a review, refer to Liu and Liu (2006). This phenomenon has been observed with volumetric organic loading rates (OLR) above 6 kg_CODs_ d^−1^ m^−3^ (Shin et al., 1992; Moy et al., 2002), with high-energy carbon sources such as carbohydrates (Morgenroth et al., 1997; Weber et al., 2007), and at higher mesophilic temperatures of 30–35°C (Weber et al., 2007; Ebrahimi et al., 2010). Filamentous overgrowth has been limited with higher up-flow mixing or aeration velocities, and with the use of acetate as carbon source (Liu and Liu, 2006). Despite the reduction of filamentous bulking with this substrate, residual filamentous structures have still been observed, and have been presumed to act as backbones for the immobilization of microbial colonies (Martins et al., 2004). In studies investigating granulation in up-flow anaerobic sludge blanket reactors, it has been observed that the same *Methanosaeta*-affiliating phylotype was constantly dominating the bacterial community during the evolution of fluffy granules to compact granules under the progressive increase in shear forces in the reactor (Grotenhuis et al., 1992; Hulshoff Pol et al., 2004). In the case of aerobic granules, analysis of bacterial compositions of fluffy and dense granules is required to assess whether different granule structures exhibit the same dominant phylotypes or not.

From the nutrient removal point of view, AGS studies initially concentrated on the formation of aerobic granules, and on the removal of organic matter (Morgenroth et al., 1997; Beun et al., 1999; Tay et al., 2002). Emphasis has then been put on achieving nitrification (Tsuneda et al., 2003), denitrification (Beun et al., 2001; Mosquera-Corral et al., 2005), dephosphatation (Lin et al., 2003), and combined BNR (de Kreuk et al., 2005; Lemaire et al., 2008; Yilmaz et al., 2008) in AGS systems. However, it has been shown that 75–100 days have been required to obtain efficient nutrient removal activities in aerobic granules after reactor start-up with flocculent activated sludge (de Kreuk et al., 2005; Xavier et al., 2007; Ebrahimi et al., 2010; Gonzalez-Gil and Holliger, 2011). In these studies, only carbon has been removed during the start-up period. Some authors have achieved enhanced granulation with faster improvements in nutrient removal performances by seeding reactors with crushed pre-cultivated granules (Pijuan et al., 2011; Verawaty et al., 2012). However, the reason why phosphorus and nitrogen removal activities have been inhibited during the first 3 months of reactor start-up with flocculent inoculation sludge and wash-out conditions has not yet been further investigated. Different microbial ecology studies have mainly been conducted on mature granules (Adav et al., 2010; Gonzalez-Gil and Holliger, 2011), but only little information is available on the microbial composition of early-stage AGS.

The present study aimed to investigate the bacterial community dynamics during the formation of early-stage aerobic granules (0–60 days) in bubble-column SBRs operated under conditions selecting for a fast-settling biomass. The main objective was to assess the effect of wash-out dynamics and operation conditions on the underlying bacterial selection, the shape of aerobic granules, the biomass settling properties, and the nutrient removal performances. We first focused on the differences in predominant bacterial populations between compact and fluffy granules, and on a way to avoid filamentous bulking in AGS systems. We then carried out a detailed monitoring of the start-up of one reactor to detect correlations between operation conditions, bacterial community dynamics and nutrient removal performances. The knowledge gained at the microbial ecology level enabled to determine why nutrient removal deteriorated during the start-up of the granulation process in bubble-column SBRs operated under wash-out conditions.

## Materials and Methods

### Reactor infrastructure and sequencing batch operation

The design and the operation of the bubble-column SBRs were adapted from de Kreuk et al. (2005). The bubble-columns consisted of internal diameters of 52–62 mm, height-to-diameter ratios of 20–25, and working volumes of 2.1–3.1 L. The SBRs were operated in fixed cycles of 3 h comprising feeding of the influent wastewater through the settled sludge bed in pulse (6 min) or anaerobic regime (60 min), aeration (110 min), biomass settling (5 min or stepwise decrease from 15 to 3 min), and withdrawal of the treated effluent (remaining cycle time). The SBRs were inoculated with 2–3 g_VSS_ L^−1^ of flocculent activated sludge originating from full-scale WWTPs. Biomass wash-out conditions were imposed with a short HRT of 6 h in order to stimulate granulation, according to Beun et al. (1999). A volume exchange ratio of 50% was applied to this end. The SBRs were operated under biomass dynamic conditions at undefined sludge retention time (SRT). The SRT was a function of the imposed settling time, of the height of the effluent withdrawal point, and of the intrinsic settling properties of the cultivated biomass. The composition of the synthetic cultivation media was similar to the one used by Ebrahimi et al. (2010), and is available in Table A1 in Appendix. Acetate was supplied as sole carbon and energy source at a constant concentration between 400 and 500 mg_CODs_ L^−1^ in the influent wastewater. This resulted in a constant volumetric OLR of 200–250 mg_CODs_ cycle^−1^ L_R_^−1^ (or 1.6–2.0 kg_CODs_ d^−1^ m_R_^−3^ daily equivalents), and in an initial biomass specific OLR of 50–60 mg_CODs_ cycle^−1^ g_CODx_^−1^ (or 0.4–0.6 kg_CODs_ d^−1^ kg_CODx_^−1^ daily equivalents). The biomass specific OLR was a dynamic function of the residual biomass concentration evolving in the reactor. The phosphorous and nitrogenous nutrient ratios amounted to 4.8 g_P-PO4_ and 12.5 g_N-NH4_ per 100 g_CODs_, respectively. During aeration, air was supplied at the target flow-rate with mass flow controllers (Brooks Instrument, Netherlands), DO was not controlled and reached saturation (8–9 mg_O2_ L^−1^), and pH was regulated at 7.0 ± 0.2 by addition of 1 M HCl or NaOH with a proportional-integral controller.

### Granulation experiments

In the first part, the granulation process was studied in five reactors (R1–R5) where the bacterial community compositions of early-stage AGS were analyzed in relation with the different combinations of operation parameters, as summarized in Table 1.

**Table 1:** **Operation parameters applied during the granulation start-up experiments**.

Reactor^1^	Inoculation sludge^2^	Temperature (°C)	Feeding regime^4^	Up-flow SAV (cm s^−1^)	Settling time^6^ (min)
R1	OMR	23 ± 2^3^	Pulse	1.8/4.0^5^	5
R2	OMR	23 ± 2^3^	Anaerobic	1.8/4.0^5^	5
R3	BNR	20	Anaerobic	1.8	15 to 3
R4	BNR	20	Anaerobic	2.0	15 to 3
R5	BNR	30	Anaerobic	1.8	15 to 3
R6	BNR	23 ± 2^3^	Anaerobic	2.5	15 to 3

*^1^R1–R5 were run in the first part of the study investigating differences in bacterial community compositions of early-stage aerobic granules. R6 was operated for detailed analysis of process conditions governing bacterial selection during granulation*.

*^2^The reactors were inoculated with flocculent activated sludge originating either from a WWTP designed for organic matter removal (OMR) only, or from a WWTP operated for full biological nutrient removal (BNR)*.

*^3^R1, R2, and R6 were operated at ambient temperature without temperature control*.

*^4^The synthetic influent wastewater was supplied either in 6 min with a pulse-feeding regime, or in 60 min with an anaerobic-feeding plug-flow regime*.

*^5^R1 and R2 were operated first with a low up-flow superficial air velocity (SAV) of 1.8 cm s^−1^. This parameter was doubled after 4 weeks for remediating filamentous overgrowth*.

*^6^Two different biomass settling patterns were tested with a constant settling time of 5 min, or with stepwise decrease in the settling time from 15 to 3 min*.

The first two reactors R1 and R2 were inoculated with a flocculent activated sludge originating from a WWTP designed for organic matter removal (OMR) only (ERM Morges, Switzerland). R1 and R2 were operated during at most 50 days at ambient temperature (23 ± 2°C), with pulse (6 min) or anaerobic plug-flow (60 min) feeding regimes, with an initially low up-flow SAV of 1.8 cm s^−1^ during aeration, and with a constant settling time of 5 min. After having observed proliferation of fluffy granules (30 days), the up-flow SAV was increased to 4.0 cm s^−1^ in order to obtain dense granules.

The reactors R3, R4, and R5 were inoculated with a flocculent activated sludge originating for a WWTP designed for full BNR along an anaerobic-anoxic-aerobic process (ARA Thunersee, Switzerland). These reactors were operated at either low (20°C, R3 and R4) or high (30°C, R5) mesophilic temperature, with anaerobic plug-flow feeding (60 min), with a low up-flow SAV of 1.8–2.0 cm s^−1^ during aeration, and with a stepwise decrease in the settling time from 15 to 3 min in 10–15 days. The operation of R4 and R5 has previously been described in detail by Ebrahimi et al. (2010), and lasted over 40 days. R3 was run on a shorter period of 15 days, but microbial ecology data were collected at higher frequency during the transition from flocculent to granular sludge.

In the second part, reactor R6 was operated with a combination of conditions selecting for the formation of dense fast-settling granules and a detailed monitoring of operation conditions, bacterial community compositions, and nutrient removal performances was carried out. R6 was inoculated with flocculent activated sludge taken from the BNR-WWTP, and was operated during 60 days at 23 ± 2°C and pH 7.0 ± 0.2, with anaerobic plug-flow feeding (60 min), a moderate up-flow SAV of 2.5 cm s^−1^, and a stepwise decrease in the settling time from 15 to 3 min. Temperature, pH, DO, and electrical conductivity signals were collected on-line. Concentrations of biomass present in the reactor and in the treated effluent, and microbial ecology data were collected on a daily basis. Liquid phase samples were taken every 3–5 days for physicochemical analyses of soluble compounds in the influent wastewater, in the reactor at the end of the anaerobic phase, and in the treated effluent.

### Characterization of metabolic activities of the inoculation sludge taken from the BNR-WWTP

The nutrient removal capacities of the inoculation sludge taken from the BNR-WWTP were tested in anaerobic, aerobic, and anoxic batch tests, and compared to the operation data of the BNR-WWTP. The tests were run at 20°C in 2-L stirred tank reactors with a biomass concentration of 3–4 g_VSS_ L^−1^ and with similar starting nutrient concentrations as in R6.

### Analyses of soluble compounds and biomass

Acetate concentration was determined with a high performance liquid chromatograph equipped with an organic acids ion exclusion column ORH-801 (Transgenomics, UK) and a refraction index detector (HPLC Jasco Co-2060 Plus, Omnilab, Germany). The concentration of anions was measured with an ICS-90 ion exchange chromatograph equipped with an IonPacAS14A column and an electrical conductivity detector (Dionex, Switzerland). The concentration of cations was measured with an ICS-3000A ion exchange chromatograph equipped with an IonPacCS16 column and an electrical conductivity detector (Dionex, Switzerland).

The particulate concentrations of total (TSS), volatile (VSS), and inorganic suspended solids (ISS) were measured according to de Kreuk et al. (2005). Granules were observed by light microscopy.

### Molecular analyses of bacterial community compositions

The compositions and dynamics of the bacterial communities were characterized by terminal-restriction fragment length polymorphism (T-RFLP) analysis targeting the v1–v3 hypervariable region of the *Eubacteria* 16S rRNA gene pool. The T-RFLP method was adapted from Rossi et al. (2009) and Ebrahimi et al. (2010), and contained the following modifications. DNA was extracted from 100 mg of homogenized biomass samples using the Maxwell 16 Tissue DNA Purification System (Promega, Switzerland). Gene fragments of 500 bp were amplified by PCR using universal eubacterial primers: a FAM-labeled 8-F forward primer (FAM-5′-GAGTTTGATCMTGGCTCAG-3′) and an unlabeled 518-R reversed primer (5′-ATTACCGCGGCTGCTGG-3′). The PCR program was run in a T3000 Thermocycler (Biometra GmbH, Germany) in 30 cycles comprising a longer denaturation time than the one used by the authors, for optimal amplification of *Accumulibacter*-related polyphosphate-accumulating organisms (PAO): 10 min initial denaturation (95°C), 1 min denaturation (95°C), 45 s primer annealing (56°C), 2 min elongation (72°C), 10 min final elongation (72°C). The amplicons were purified and concentrated using Invisorb MSB Spin PCRapace purification kits (Invitek Stratec Molecular GmbH, Germany). Amounts of 200 ng of purified PCR products were digested at 37°C for 3 h with 0.5 units of the *Hae*III endonuclease (Promega, Switzerland). Volumes of 1 μL of digestion products were mixed with 8.5 μL of HiDi formamid and 0.5 μL of GeneScan 600-LIZ internal size standard (Applied Biosystems, USA), and were denaturated for 2 min at 95°C. The terminal-restriction fragments (T-RFs) were separated and analyzed by capillary gel electrophoresis in an ABI Prism 3100-Avant Genetic Analyzer using a fluid POP-6 gel matrix and fluorescence laser detection (Applied Biosystems, USA). The T-RFLP profiles were aligned using the Treeflap crosstab macro (Rees et al., 2004). The bacterial community structures were expressed as relative contributions of all operational taxonomic units (OTU) contributing to the total measured fluorescence. Predominant OTUs with relative abundances above 2% were presented in stacked bar plots for simplified visual observation. Three single biomass samples from the whole set of samples of the study were analyzed in triplicates to determine the overall relative standard deviation related to the T-RFLP method (6%). For reactor R6, biomass equivalents of target OTUs were expressed by multiplying their relative abundances by the mass of VSS present in the reactor.

### Analysis of the richness and diversity of the bacterial community evolving in reactor R6

Richness and Shannon’s H’ diversity indices were computed with the R software version 2.14.1 equipped with the Vegan package (R-Development-Core-Team, 2008; Oksanen et al., 2009) based on the full T-RFLP profiles collected during experiment R6. Mathematical geometric evolution models were fitted to the measured richness and diversity profiles with the Berkeley Madonna software (Macey et al., 2000) in order to simulate the evolution of both indices in the reactor. Standard deviation intervals on model predictions were computed from 1000 Monte Carlo simulations on underlying parameters.

### Phylogenetic affiliation of operational taxonomic units

Predominant OTUs detected in R1–R5 were affiliated to closest bacterial relatives by using the cloning-sequencing databank developed by Ebrahimi et al. (2010) and complemented in the present study. Two DNA extracts from biomass samples collected in R6 at day 2 (flocculent sludge) and day 59 (granular sludge) were sent to Research and Testing Laboratory (Lubbock, TX, USA) for 454 Tag-encoded FLX amplicon pyrosequencing with a Genome Sequencing FLX System (Roche, Switzerland) using the procedure developed by Sun et al. (2011), and the same primers (8-F and 518-R) as the ones used for T-RFLP analysis. The pyrosequencing datasets were denoised and processed with the PyroTRF-ID bioinformatics procedure developed by Weissbrodt and Shani, et al. (paper submitted), which includes sequence annotation with the Greengenes database (McDonald et al., 2012), digital T-RFLP profiling, comparison of digital and experimental T-RFLP profiles, and phylogenetic affiliation of OTUs. QIIME algorithms were used in the denoising process (Caporaso et al., 2010).

### Bacteriome analysis

The pyrosequencing datasets of the two biomass samples collected in R6 were analyzed by the metagenomics RAST server (MG-RAST; Meyer et al., 2008) for annotation and comparative analysis. The Ribosomal Database Project (RDP; Cole et al., 2009) was used as sequence annotation source. A minimum identity cutoff of 97% was applied in order to retain only the closest bacterial affiliations. A circular phylogenetic tree was constructed with the pyrosequencing datasets of the two samples. The tree was complemented with two bar plots representing the relative abundances of the bacterial genera in both samples. Richness and Shannon’s H’ diversity indices were also computed from these datasets.

## Results

### Composition and activity of early-stage granules cultivated from flocculent OMR-sludge

**Figure 1:**
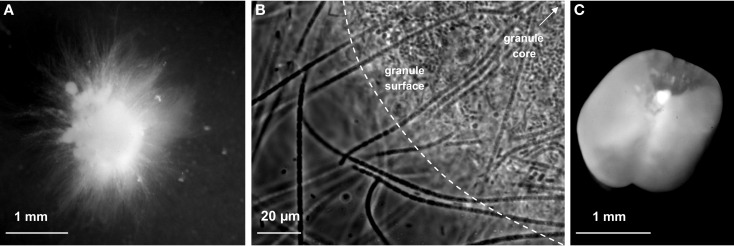
**Example of early-stage aerobic granule structures observed with light microscopy**. Fluffy slow-settling granule obtained after 30 days in reactor R2 with OMR-sludge and low up-flow SAV of 1.8 cm s^−1^, and exhibiting filamentous outer structures **(A)**. Filamentous segmented chain bacterial structures interspersing across the granular biofilm observed on a sample collected on day 22 in R2 **(B)**. Dense fast-settling granule present after 50 days in R6 with BNR-sludge and moderate up-flow SA of 0.025 m s^−1^, and displaying a tulip-like folded structure around a more opaque internal core **(C)**.

**Figure 2:**
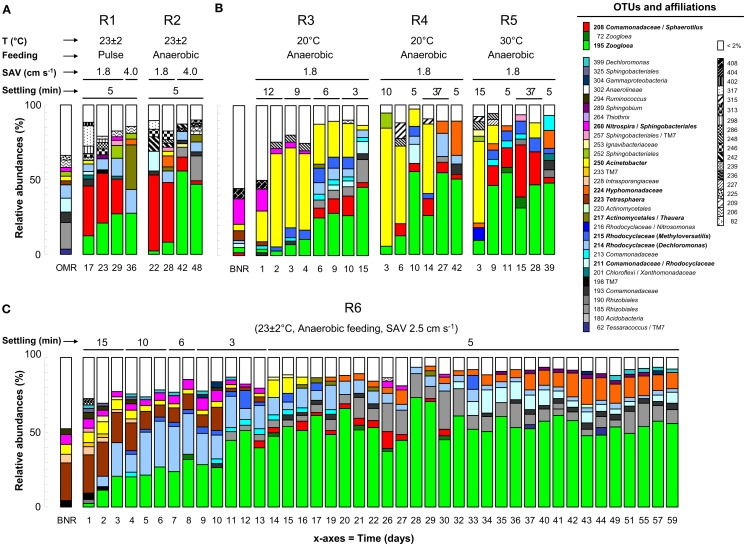
**Dynamics of predominant bacterial OTUs analyzed with T-RFLP during the six granulation experiments**. Reactors R1 and R2 were inoculated with activated sludge from the OMR-WWTP **(A)**. R3, R4, and R5 were inoculated with activated sludge from the BNR-WWTP **(B)**. High resolution bacterial ecology data were collected from R6 to assess the effect of wash-out dynamics on bacterial selection during granulation **(C)**. Main operation conditions are indicated at the top of each graph. Closest bacterial affiliations of target OTUs presented in Table 2 are given on the right.

Reactors R1 and R2 were inoculated with flocculent activated sludge taken from an aeration tank of a WWTP designed for OMR only (OMR-sludge). Operation at ambient temperature (23 ± 2°C), with acetate as carbon source, a low up-flow SAV of 1.8 cm s^−1^, and a fixed settling time of 5 min resulted in the proliferation of slow-settling fluffy early-stage granules (Figure 1A). Segmented chain filamentous bacterial structures were detected by light microscopy (Figure 1B). The underlying bacterial community compositions are presented in Figure 2A. Closest bacterial affiliations of specific OTUs detected in these reactors are given in Table A2 in Appendix. The fluffy granules obtained in R1 with pulse feeding (6 min) were predominantly composed of *Burkholderiales* affiliates related to the *Sphaerotilus*-*Leptothrix* group (OTU-208, 23–33%), and by *Zoogloea* spp. belonging to *Rhodocyclales* (OTU-195, 12–27%). In R2 operated with anaerobic feeding (60 min), *Sphaerotilus*-*Leptothrix* affiliates dominated (39–50%) over *Zoogloea* spp. (2–8%) in fluffy granules.

**Table 2:** **Closest phylogenetic bacterial affiliations of specific OTUs detected in reactor R6, that were obtained after pyrosequencing, mapping, and digital T-RFLP profiling with the PyroTRF-ID bioinformatics procedure (Weissbrodt and Shani et al., paper submitted)**.

T-RF^1^(bp)	Sample^2^	Counts^3^ (−)	Fraction of T-RF^4^ (%)	Affiliation^5^		Accession number^5^	Closest relative and original microbiota^6^	Smith-Waterman mapping score (−)^7^	
				Phylum → Order	Family → Genus			Absolute	Normalized
62	Day 2	22	67	O: *Actinomycetales*	G: *Tessaracoccus*	GQ097568	Uncultured bacterium clone nbw397a07c1 from human skin microbiome	380	0.977
		10	30	P: candidate phylum TM7		EU104134	Uncultured bacterium clone M0509_49 from activated sludge	373	0.912
72	Day 2	18	40	O: *Rhodocyclales*	G: *Zoogloea*	AJ011506	*Zoogloea resiniphilia* strain DhA-35T	376	0.962
		13	35	O: *Rhodocyclales*	G: *Thauera*	AY945909	Uncultured bacterium clone DR-48 from denitrifying bioreactor	348	0.926
	Day 59	11	100	O: *Rhodocyclales*	G: *Zoogloea*	AJ011506	*Zoogloea resiniphilia* strain DhA-35T	373	0.942
180	Day 2	4	100	P: *Acidobacteria*		GQ396818	Uncultured bacterium clone AK1AB1_04A from recently deglaciated soils	323	0.807
185	Day 2	8	89	O: *Rhizobiales*		FJ719048	Uncultured bacterium clone p03_D09 from aquifer sediments	296	0.757
		1	11	O: *Rhodobacterales*		FJ719099	Uncultured bacterium clone p04_H05 from aquifer sediments	318	0.941
	Day 59	14	100	O: *Rhizobiales*		AF502218	Uncultured bacterium clone HP1B02 from EBPR sludge	409	0.969
190	Day 2	20	49	O: *Rhizobiales*		CU918969	Uncultured *Alphaproteobacterium* clone QEEA1DD02 from anaerobic digester	404	0.962
		18	44	O: *Burkholderiales*	G: *Acidovorax*	EU539550	Uncultured bacterium clone nbt241e12 from human skin microbiota	356	0.944
193	Day 2	22	30	O: *Burkholderiales*	G: *Acidovorax*	EU375646	*Acidovorax* sp. u41 from bacterial community degrading organic pollutants	363	0.936
		21	28	O: *Burkholderiales*	G: *Simplicispira*	AJ505861	*Comamonadaceae* bacterium strain PIV-16-2 from denitrifying bacterial community	377	0.969
		13	17	O: *Burkholderiales*	G: *Delftia*	EF515241	Uncultured bacterium clone 21g10 from electricigen enrichment in a MFC	355	0.920
	Day 59	72	94	O: *Burkholderiales*	G: *Acidovorax*	AJ864847	*Acidovorax* sp. strain J33 from high mountain lake habitats	383	0.923
		5	6	O: *Xanthomonadales*		EU662583	Uncultured bacterium clone MC1_16S_13 from sulfidic karst system	343	0.868
195	Day 2	231	84	O: *Rhodocyclales*	G: *Zoogloea*	AF234684	Uncultured sludge bacterium H7 from nitrifying-denitrifying industrial WWTP	385	0.990
		18	7	O: *Pseudomonadales*	G: *Acinetobacter*	GQ073520	Uncultured bacterium clone nbw209f04c1 from human skin microbiome	337	0.988
	Day 59	4793	100	O: *Rhodocyclales*	G: *Zoogloea*	EU639144	Uncultured bacterium clone LPB19 from EBPR sludge	402	0.854
198	Day 2	65	94	P: candidate phylum TM7		DQ640696	Uncultured TM7 bacterium clone Skagenf80 from EBPR-WWTP	367	0.870
201	Day 2	10	48	P: *Chloroflexi*		CU927307	Uncultured *Chloroflexi* bacterium clone EDN7BB07 from anaerobic digester	443	1.000
		8	38	O: *Xanthomonadales*	F: *Xanthomonadaceae*	FJ612198	Uncultured bacterium clone DP3.5.36 from lake ecosystem	360	0.923
208	Day 2	6	30	O: *Burkholderiales*	G: *Rhodoferax*	AB154311	Uncultured bacterium clone S9F-53 from eutrophic lake	351	0.931
		1	10	O: *Burkholderiales*	G: *Sphaerotilus*	AB087568	*Sphaerotilus* sp. L19 from filamentous activated sludge bulking process	374	0.979
210	Day 2	6	42	O: *Acidobacteriales*		FJ230900	Uncultured bacterium clone F25 from river water receiving antibiotics-rich effluents	403	0.988
		5	35	P: *Firmicutes*	G: *Trichococcus*	EU234209	Uncultured bacterium clone B14 from river water receiving antibiotics-rich effluents	295	1.000
211	Day 59	7	54	O: *Burkholderiales*	F: *Comamonadaceae*	EF540425	Uncultured soil bacterium clone MK4a from semi-coke	345	0.925
		6	46	O: *Rhodocyclales*	F: *Rhodocyclales*	DQ088735	Uncultured bacterium clone BE16FW031601GDW_hole1-9 from gold mine groundwater	343	0.724
213	Day 2	17	73	O: *Burkholderiales*	F: *Comamonadaceae*	AY662010	Uncultured bacterium clone 300A-D08 from groundwater contaminated with nitric acid	365	0.979
214	Day 2	136	48	O: *Rhodocyclales*	G: *Dechloromonas*	AY064177	Uncultured *Betaproteobacterium* clone UCT N123 from EBPR-WWTP	382	0.977
		45	16	O: *Burkholderiales*	G: *Rhodoferax*	AB452981	*Betaproteobacterium* clone HIBAF001 from freshwater bacterioplankton	366	0.948
		29	10	O: *Rhodocyclales*	G: *Methyloversatilis*	AY436796	*Methyloversatilis universalis* strain EHg5 isolated from sediments	364	0.958
		12	4	O: *Rhodocyclales*	G: *Zoogloea*	DQ413172	*Zoogloea* sp. EMB 357 isolated from anaerobic-aerobic SBR	344	0.901
		11	4	O: *Burkholderiales*	G: *Aquamonas*	DQ521469	Uncultured bacterium clone ANTLV1_A07 from Antarctica lake ice cover microbiota	366	0.951
		4	1	O: *Rhodocyclales*	G: *Rhodocyclus*	EF565151	Uncultured bacterium clone VIR_D5 from EBPR sludge rich in *Accumulibacter*	368	0.981
	Day 59	14	88	O: *Rhodocyclales*	G: *Dechloromonas*	DQ413103	Uncultured bacterium clone 44 from anaerobic-aerobic SBR	381	0.890
		2	12	O: *Rhodocyclales*	G: *Rhodocyclus*	AF502224	Uncultured bacterium clone HP1A13 from EBPR sludge	372	0.923
215	Day 2	14	44	O: *Rhodocyclales*	G: *Methyloversatilis*	DQ066958	Uncultured bacterium clone pLW-7 from sediment consortium metabolizing C1 compounds	349	0.928
		9	28	P: *Chloroflexi*	G: *Caldilinea*	CU917747	Uncultured *Chloroflexi* bacterium clone QEEB2DA06 from anaerobic digester	279	0.730
		2	6	O: *Rhodocyclales*	G: *Rhodocyclus*	FJ719063	Uncultured bacterium clone p04_A04 from aquifer sediments	320	0.938
		1	3	O: *Rhodocyclales*	G: *Dechloromonas*	AY062126	Uncultured *Betaproteobacterium* clone UCT N141 from EBPR-WWTP	306	0.820
216	Day 2	5	35	O: *Rhodocyclales*	F: *Rhodocyclales*	NR029035	*Quatrionicoccus australiensis* strain Ben 117 from activated sludge	311	0.881
		3	21	O: *Burkholderiales*	F: *Comamonadaceae*	EU180529	*Betaproteobacterium* BAC49 from granular activated carbon filters	273	0.853
		1	7	O: *Nitrosomonadales*	G: *Nitrosomonas*	EU937892	Uncultured bacterium clone 3BR-6DD from an iron oxidizing freshwater habitat	348	0.909
217	Day 2	15	46	O: *Actinomycetales*		AF513101	Uncultured bacterium clone 7 from foaming activated sludge	385	0.955
		12	36	O: *Rhodocyclales*	G: *Thauera*	AM084110	*Thauera* sp. R-28312 from denitrifying sludge	386	1.000
223	Day 2	545	99	O: *Actinomycetales*	G: *Tetrasphaera*	AF255629	Uncultured bacterium clone Ebpr19 from EBPR-WWTP	374	0.944
	Day 59	23	100	O: *Actinomycetales*	G: *Tetrasphaera*	AF527583	Uncultured bacterium clone LPB21 from EBPR sludge	371	0.949
224	Day 59	135	96	O: *Rhodobacterales*	F: *Hyphomonadaceae*	AF236001	*Alphaproteobacterium* A0904	285	0.625
228	Day 2	50	88	O: *Actinomycetales*	F: *Intrasporangiaceae*	AF513091	Uncultured bacterium clone 17 from faming activated sludge	382	0.946
		3	5	C: *Actinobacteria*	F: *Microthrixaceae*	CU917839	Uncultured *Actinobacterium* clone QEEB1AC11 from anaerobic digester	388	0.965
233	Day 2	26	87	P: candidate phylum TM7		FJ534960	Uncultured bacterium clone 14 from anaerobic fermentation of waste activated sludge	271	0.666
		2	7	O: *Phycisphaerales*		FJ612210	Uncultured bacterium clone DP7.3.10 from lake ecosystem	283	0.625
250	Day 2	35	92	O: *Pseudomonadales*	G: *Acinetobacter*	EU467673	Uncultured bacterium clone molerat_2g12_1 from gut microbiota	415	0.883
252	Day 2	4	80	O: *Sphingobacteriales*		FJ793188	Uncultured bacterium clone TDB87 from a hot spring dam	295	0.905
253	Day 2	14	88	O: *Ignavibacteriales*	F: *Ignavibacteriaceae*	AB186808	Uncultured bacterium from polychlorinated-dioxin-dechlorinating microbial community	462	0.977
257	Day 2	7	58	O: *Sphingobacteriales*		EF562554	Uncultured bacterium clone CC_3 from consortium degrading complex organic matter	380	0.997
		5	42	P: candidate phylum TM7		AB200304	Uncultured bacterium clone UTFS-OF09-d22-29 from EBPR-WWTP	283	0.663
260	Day 2	16	76	O: *Nitrospirales*	G: *Nitrospira*	AF314422	Uncultured bacterium PHOS-HE34 from an aerobic EBPR ecosystem	366	0.924
		4	19	O: *Sphingobacteriales*		FJ660602	Uncultured bacterium clone A194 from full-scale WWTP	334	0.859
	Day 59	3	100	O: *Sphingobacteriales*		AY302128	Uncultured bacterium clone DSBR-B082 from denitrifying community	354	0.878
264	Day 59	3	100	O: *Thiotrichales*	G: *Thiothrix*	L79963	*Thiothrix fructosivorans* strain I, a filamentous sulfur bacterium from WWTP	334	0.933
289	Day 2	4	57	O: *Sphingomonadales*	G: *Sphingobium*	AB040739	*Sphingobium* cloacae a non-ylphenol-degrading bacterium isolated from WWTP	277	0.785
		3	43	O: *Rhodospirillales*	F: *Rhodospirillaceae*	EU864465	Uncultured bacterium clone E52 from river water receiving antibiotics-rich effluents	350	0.967
294	Day 2	1	100	O: *Clostridiales*	G: *Ruminococcus*	DQ796981	Uncultured bacterium clone RL386_aao85c11 from human gut microbiome	289	0.906
302	Day 2	6	75	C: *Anaerolineae*		EU332818	Uncultured organism clone OTUI177 from aerobic EBPR-SBR	310	0.831
304	Day 2	28	93	C: *Gammaproteobacteria*		FJ356049	Uncultured bacterium clone G5 from lab-scale EBPR system	383	0.844
325	Day 2	4	100	O: *Sphingobacteriales*		GQ396974	Uncultured bacterium clone AK1DE1_04E from recently deglaciated soils	299	0.779
399	Day 59	12	100	O: *Rhodocyclales*	G: *Dechloromonas*	EF632559	*Dechloromonas* sp. A34, a bacterium from phosphate mining overburden respiring selenate	378	0.922

*^1^Size of target terminal-restriction fragments (T-RF) obtained with *Hae*III digestion and forming operational taxonomic units (OTU)*.

*^2^Original biomass sample selected for pyrosequencing analysis*.

*^3^Number of sequences from the pyrosequencing dataset that were related to the particular reference organism*.

*^4^Different bacterial populations can contribute to the same T-RF. The percentage of contribution of each population to the target T-RF is given in this column*.

*^5^Closest bacterial affiliations and GenBank accession numbers obtained after mapping against the Greengenes reference sequences of 16S rRNA encoding gene (McDonald et al., 2012). Legend: P, phylum; C, class; O, order; F, family; G, genus*.

*^6^Description of closest relatives and original microbiota from which the reference clones were isolated were obtained after submitting the accession numbers into the GenBank public database (Benson et al., 2011)*.

*^7^The Smith–Waterman (SW) score was used as mapping similarity measure. SW scores consider nucleotide positions and gaps in the sequence structures. The highest absolute SW score that can be obtained is the length of the sequence itself. Each absolute SW score was normalized to the length of the related denoised centroid sequence in order to allow comparison between sequences of various lengths. After mapping in MG-RAST (Meyer et al., 2008), the affiliations obtained for the two denoised pyrosequencing datasets were related to traditional sequence identity scores of 99.7 ± 0.5%*.

The application of a higher up-flow SAV of 4.0 cm s^−1^ after 30 days resulted in the recovery of smooth and dense fast-settling granules. The predominant organisms shifted from *Sphaerotilus*-*Leptothrix* affiliates to *Zoogloea* spp. In the granules of R1, *Zoogloea* spp. (27%) and *Thaurea* spp. (OTU-217, 30%) outcompeted *Burkholderiales* (<1%). The dense granules of R2 were highly dominated by *Zoogloea* spp. (47–57%). *Burkholderiales* decreased to 2% after day 48. OTU-185 affiliating with *Gammaproteobacteria* and with *Rhizobiales* from *Alphaproteobacteria* was present up to 17%.

At physical reactor boundaries, filamentous bulking led to deteriorated sludge settling. Both reactors displayed poor nutrient removal performances. After the recovery of fast-settling granules, nutrient removal did not improve. After pulse feeding in R1, acetate was fully removed within 40 min during the aeration phase. In R2 where slow plug-flow anaerobic feeding was applied, more than 90% of the acetate leaked into the aeration phase during which it was fully consumed. Ammonium was not nitrified and biological dephosphatation did not occur. Only partial nitrogen (20%) and phosphorus removal (10%) was detected in both reactors which was probably due to anabolic requirements.

### Composition and activities of early-stage granules cultivated from flocculent BNR-sludge

The reactors R3, R4, and R5 were operated with an inoculation sludge originating from a WWTP designed for full BNR, under anaerobic feeding, and with a stepwise decrease in the settling time from 15–3 min. The three reactors resulted in the formation of smooth and dense fast-settling granules after 9–10 days (Figure 1C). The underlying bacterial community dynamics are presented in Figure 2B. The inoculation sludge from the BNR-WWTP was dominated by *Nitrospira* and *Sphingobacteriales* (OTU-260, 17%), *Tetrasphaera* spp. (OTU-223, 7%), and an unidentified OTU-408 (7%). *Zoogloea* spp. (OTU-195), *Burkholderiales* (OTU-207), OTU-210 affiliating with *Acidobacteriales* and *Firmicutes*, and OTU-214 affiliating with *Rhodocyclales*-related organisms such as *Dechloromonas* and *Methyloversatilis* spp. were present in lower abundances (2–3%). In all three reactors, the sludge was still in the flocculent state over the first 6 days. The predominant organisms of the inoculum were replaced within 2–3 days by *Acinetobacter* spp. (OTU-250, 21–79%). Granulation correlated with the proliferation of *Zoogloea* spp. (28–55%). *Dechloromonas* (5–16%), *Methyloversatilis* (3–10%), and *Rhizobiales* (4–16%) were detected as flanking populations. *Hyphomonadaceae* affiliates were abundantly present after 42 days in R4 (23%), and after 39 days in R5 (12%). In contrast to the operation at 20°C in R3 and R4 where dense fast-settling granules were constantly present, the operation at 30°C in R5 led to the proliferation of organisms affiliated to the *Sphaerotilus*-*Leptothrix* group (35%) and resulted in a mixture of dense and fluffy granules. Even though a BNR-sludge was used as inoculum, nitrification and dephosphatation activities were not detected in the three AGS systems. Acetate was only consumed to a small extent during the anaerobic feeding phases (18–25%) and fully removed during the aeration phases.

### Dynamics of bacterial communities and process performance under wash-out conditions

**Figure 3:**
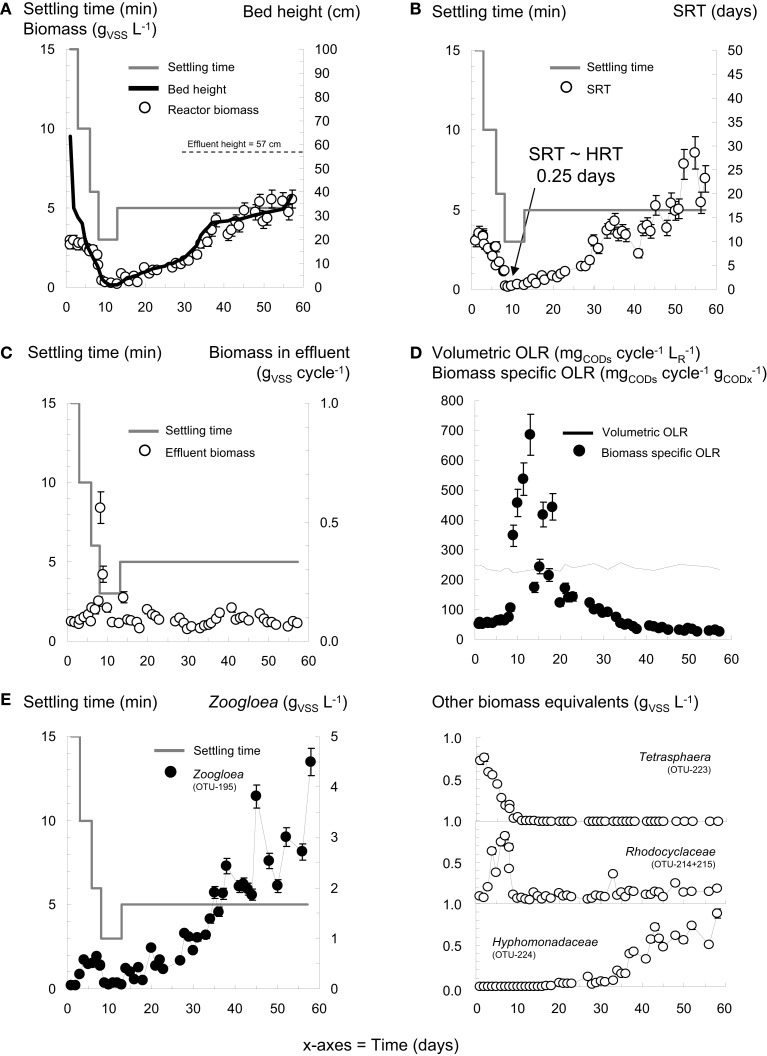
**Detailed evolution of biomass parameters during granulation in reactor R6 under wash-out dynamics, in function of the imposed settling time**. The evolution of the biomass concentration and of the height of the settled sludge blanket displayed similar profiles as soon as the settling time was decreased to 3 min **(A)**. The application of wash-out conditions resulted in the re-coupling of the SRT to the HRT at day 8 **(B)**. A high amount of biomass was withdrawn with the effluent wastewater during wash-out from day 8 on **(C)**. Under reactor operation with a constant volumetric OLR, the biomass specific OLR exhibited a drastic increase during the period of extensive wash-out between day 8 and day 20 **(D)**. *Zoogloea*, *Tetrasphaera*, *Rhodocyclaceae*, and *Hyphomonadaceae* affiliates displayed distinct biomass evolutions **(E)**. Early-stage granules nuclei formed after 10 days.

For reactor R6, high frequency of data collection allowed to detect correlations between operation conditions, bacterial dynamics, and BNR performances during early-stage granulation under wash-out conditions. Changes in biomass properties are presented in Figures 3A,B in function of the settling time. With initial settling times of 15 and then 10 min during the first 5 days, the activated sludge remained in the flocculent state and a biomass concentration of 2.45–2.95 g_VSS_ L^−1^ was maintained in the reactor, forming a settled sludge blanket of 15–30 cm, and the SRT amounted to 12 days. The decrease in the settling time from 6 to 3 min at day 8 resulted in extensive biomass wash-out (Figure 3C). An extremely low residual biomass concentration of 0.2 g_VSS_ L^−1^ was remaining in the system, and formed a settled sludge blanket of only 1 cm. The SRT dropped to 0.5 day, and approached the HRT of 0.25 day. First granule nuclei were observed after 10 days. At day 12, the settling time was increased to 5 min as safety measure to keep the granules in the system. The granular biomass increased to 4.0 g_VSS_ L^−1^ at day 37, progressively stabilized at 5.3 ± 0.4 g_VSS_ L^−1^ after 52 days, and formed a settled sludge blanket of 32–40 cm. The fraction of ISS amounted to 38% in the inoculation sludge, and to 12% in the early-stage AGS. An example of a dense fast-settling granule present in R6 after 50 days is presented in Figure 1C.

The reactor was operated with a constant volumetric OLR of 250 mg_CODs_ cycle^−1^ L_R_^−1^. The biomass specific OLR however evolved with the residual biomass concentration from initially 51 to 685 mg_CODs_ cycle^−1^ g_CODx_^−1^ between day 8 and day 20 after the intensive biomass wash-out (Figure 3D). As the AGS biomass grew in the system, the biomass specific OLR progressively decreased to 20 mg_CODs_ cycle^−1^ g_CODx_^−1^.

*Tetrasphaera* spp. (OTU-223) dominated in the inoculation sludge (26%), and were progressively replaced in the flocculent sludge after 5 days by *Zoogloea* spp. (OTU-195, 21%) and by OTU-214 (29%; Figure 2C). During this initial phase, mainly *Dechloromonas* and *Comamonadaceae* relatives contributed to OTU-214, whereas *Accumulibacter* accounted for only 1% of this OTU (Table 2). When expressed as biomass concentration equivalents, OTU-195 and OTU-214 increased during this period up to 0.6 and 0.8 g_VSS_ L^−1^, respectively (Figure 3E). The extensive biomass wash-out at day 8 resulted in the rapid decrease in the masses of all bacterial populations below 0.1 g_VSS_ L^−1^. *Zoogloea* spp. then rapidly proliferated up to a relative abundance of 54 ± 8% in the early-stage AGS from day 15 to day 60. Other *Rhodocyclales* affiliates (*OTUs* 214 and 215) declined below 5% at day 26. The concentration of *Zoogloea* spp. amounted to 3.0 g_VSS_ L^−1^ after 52 days. The concentration of other *Rhodocyclales* affiliates remained low, but exhibited a slight increase from 0.06 to 0.19 g_VSS_ L^−1^ from day 10 to day 60.

After granulation, additional bacterial populations evolved in the AGS. The relative abundance of *Rhizobiales* (OTU-185) increased from 6% at day 11 to 26% at day 39, and stabilized subsequently at 10 ± 4% over the next 20 days. *Hyphomonadaceae* (OTU-224) were detected above 1% from day 17 on, and were present at 13 ± 3% after day 37. *Comamonadaceae* (OTU-211) increased up to 16% at day 34, and remained at 5 ± 2% until the end of the experiment. *Acinetobacter* spp. (OTU-250) were only detected during the first 17 days in relative abundances of 3–12%. OTU-260 affiliating with *Sphingobacteriales* (and *Nitrospira* probably to a less extent) was present up to 7% at day 10, but was only present at low relative abundances of <1–4% in the early-stage granules. Nitrifiers were not detected above the detection limit of the T-RFLP method.

**Figure 4:**
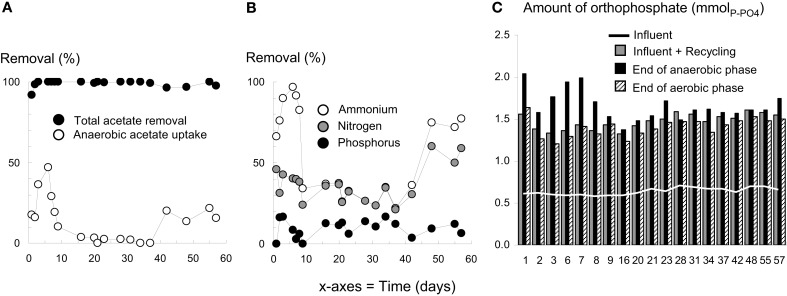
**Detailed evolution of the nutrient removal performances in reactor R6**. The application of wash-out dynamics resulted in the transient loss of anaerobic acetate uptake **(A)**, nitrification, nitrogen removal, and phosphorus removal performances **(B)** from day 6 to day 40. Orthophosphate cycling activities in alternating anaerobic-aerobic conditions were not detected during the same period **(C)**.

Wastewater treatment plants operation data and metabolic batch tests indicated that the inoculation sludge was efficiently removing organic matter (95%), nitrogen (97%), and phosphorus (92%). BNR activities were detected in R6 during initial operation with a high settling time (Figures 4A,B). After 6 days, 48% of the acetate load was consumed during anaerobic feeding, ammonium was efficiently nitrified to nitrate (97%), and 40% of nitrogen was removed. Two millimoles of orthophosphate were cycled in alternating anaerobic-aerobic conditions (Figure 4C), but only 9% of phosphorus was effectively removed. After intensive biomass wash-out at day 8, BNR activities were lost except carbon removal. Between day 10 and day 40, less than 4% of acetate was taken up during the anaerobic feeding phase, and only 31 ± 6% of ammonium was removed, presumably by assimilation into biomass. The orthophosphate cycling activity was lost, and phosphorus removal remained at 11 ± 4% until the end of the experiment. After day 40, ammonium and nitrogen removal recovered to 77 and 60%, respectively. However, nitrite instead of nitrate accumulated in the system. A slight increase in the anaerobic acetate uptake (up to 22%) was detected.

### Bacteriome analysis of the flocculent and granular sludge in R6

**Figure 5:**
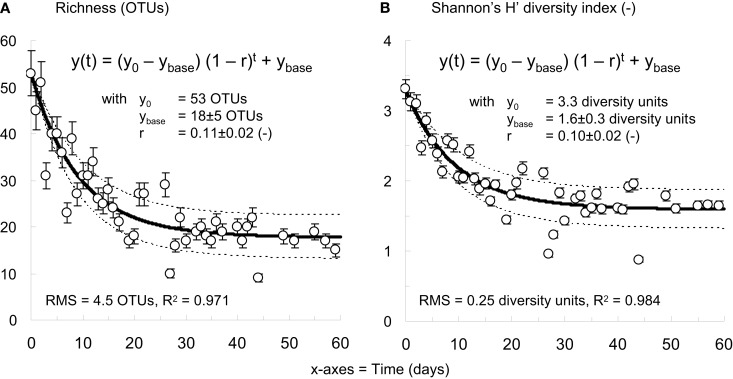
**The bacterial community present in R6 exhibited a strong decrease of about 66% in richness (A) and of about 52% in Shannon’s H’ diversity (B) from inoculation with flocculent activated sludge from a full-scale BNR-WWTP fed with real wastewater to formation of early-stage granules fed with an acetate-based synthetic influent**. Mathematical negative exponential growth models successfully explained the evolution of both indices during reactor start-up (*R*^2^ = 0.97–0.98). The model trends are given with standard deviation intervals computed from 1000 Monte Carlo simulations. Legend: y_0_: initial richness or diversity value, y_base_: average final richness or diversity value after 60 days, r: negative growth rate, RMS: root mean square error.

Based on the T-RFLP data collected from R6, the bacterial community was displaying a strong decrease of 66% in richness and 52% in diversity during the start-up of the reactor (Figure 5). The bacterial community of the activated sludge taken from the full-scale BNR was associated with a richness of 53 OTUs and a diversity index of 3.3. The bacterial community of the early-stage granules (day 30–60) was composed of 18 ± 5 OTUs, and showed a diversity index of 1.6 ± 0.3. The best fits of the mathematical geometric evolution models to the evolution of richness and diversity indices (R^2^ = 0.97 and 0.98, respectively) were obtained with finite rates of decrease of 11 and 10%, respectively. According to the models, the decrease in richness and diversity before extensive biomass wash-out (day 0–8) amounted to 38 and 30%, i.e., apparent decrease rates of about 2.5 OTUs and 0.13 diversity units per day. During the formation of early-stage granules occurring after biomass wash-out (day 8–27), the richness and diversity decreased by another 27 and 21% (0.8 OTUs and 0.04 diversity units per day).

The pyrosequencing analyses of biomass samples collected on day 2 and day 59 confirmed that the early-stage AGS displayed a strongly reduced richness and diversity compared to the initial flocculent sludge (Table 3). The bacteriome of the flocculent sludge on day 2 was composed of 50 orders and 170 genera that were evenly distributed (3.9 diversity units). The bacteriome of the early-stage AGS was composed of only 20 orders and 57 genera that were unevenly distributed (1.1 diversity units). This analysis also showed that the T-RFLP method was covering at least 85% of the diversity obtained by pyrosequencing. Within the *Rhodocyclales* order, *Zoogloea* affiliates became very predominant in the early-stage AGS, and *Dechloromonas*-related organisms that were abundant in the flocculent sludge at day 2 were replaced by *Accumulibacter* and *Azoarcus* relatives in the early-stage AGS at day 59.

**Table 3:** **Summary of the main bacterial orders and genera identified by pyrosequencing analysis of the flocculent sludge and early-stage AGS samples taken from R6**.

Main bacterial orders	%^1^	Main bacterial genera and relative abundances
**FLOCCULENT SLUDGE (DAY 2)**		
*Rhodocyclales*	24	*Zoogloea* (10%), *Dechloromonas* (9.7%), *Methyloversatilis* (1.4%), *Azoarcus* (1.3%), *Thauera* (1.2%)
*Actinomycetales*	10	*Tetrasphaera* (3.4%), *Terrabacter* (3.2%), *Nocardia* (1.0%), *Micrococcus* (0.4%), *Streptomyces* (0.3%)
*Burkholderiales*	9	*Acidovorax* (3.7%), *Diaphorobacter* (1.4%), *Burkholderia* (1.2%), *Alcaligenes* (0.7%), *Hydrogenophaga* (0.4%)
*Pseudomonadales*	9	*Acinetobacter* (8.0%), *Pseudomonas* (0.7%)
*Bacillales*	6	*Brevibacillus* (5.3%), *Trichococcus* (0.3%)
*Chromatiales*	5	*Thiolamprovum* (1.7%), *Allochromatium* (1.7%), *Halochromatium* (0.7%)
*Rhodobacterales*	5	*Azospirillum* (2.0%)
*Rhodospirillales*	3	*Rhodobacter* (3.7%), *Rhodobaca* (0.7%)
*Rhizobiales*	2	*Methylosinus* (1.1%), *Rhodopseudomonas* (0.5%), *Methylocystis* (0.3%), *Bradyrhizobiaceae* (0.1%)
*Sphingobacteriales*	2	*Terrimonas* (1.3%), *Chitinophaga* (0.9%)
*Sphingomonadales*	2	*Sphingomonas* (1.8%)
*Bacteroidales*	2	*Butyricimonas* (2.0%)
38 residual orders (<2%)	21	137 residual genera
**EARLY-STAGE AGS (DAY 59)**		
*Rhodocyclales*	84	*Zoogloea* (80%), *Accumulibacter* (3.6%), *Azoarcus* (2.8%), *Thauera* (0.6%)
*Burkholderiales*	4	*Massilia* (2.5%), *Comamonas* (0.5%), *Acidovorax* (0.5%)
*Flavobacteriales*	3	*Flavobacterium* (2.8%)
*Xanthomonadales*	2	*Stenotrophomonas* (1.0%), *Pseudoxanthomonas* (0.3%), *Dyella* (0.2%)
*Neisseriales*	2	*Aquitalea* (1.4%)
15 residual orders (<2%)	5	45 residual genera

**Figure A1:**
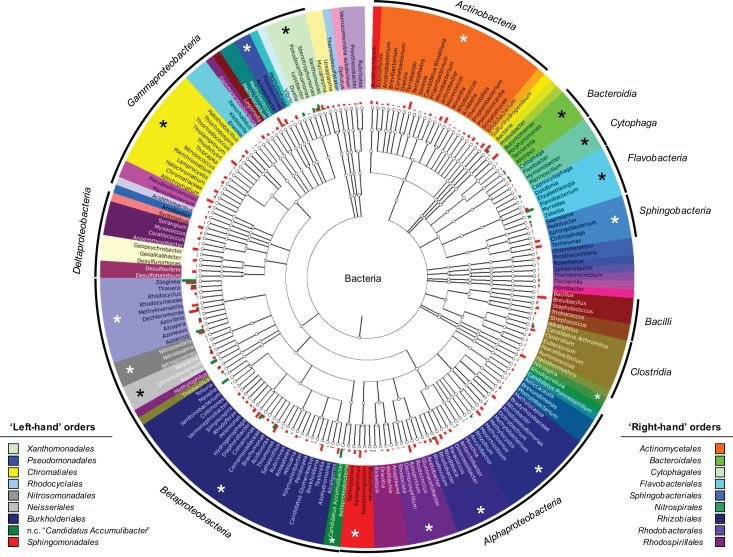
**Bacteriome phylogenetic tree constructed in MG-RAST (Meyer et al., 2008) with the pyrosequencing datasets of the two biomass samples collected on day 2 (flocculent sludge, red bar plot) and day 59 (early-stage AGS, green bar plot) in the R6 reactor**. Each bar plot is related to the number of pyrosequencing reads detected per bacterial affiliation. The tree is presented with classes (outer black circle segments) and orders subdivisions (colored slices), and bacterial genera names. The identity of target orders marked with an asterisk is given for each left-hand and right-hand half of the circular tree. The RDP database (Cole et al., 2009) was used as annotation source, and a minimum identity cutoff of 97% was applied.

**Figure A2:**
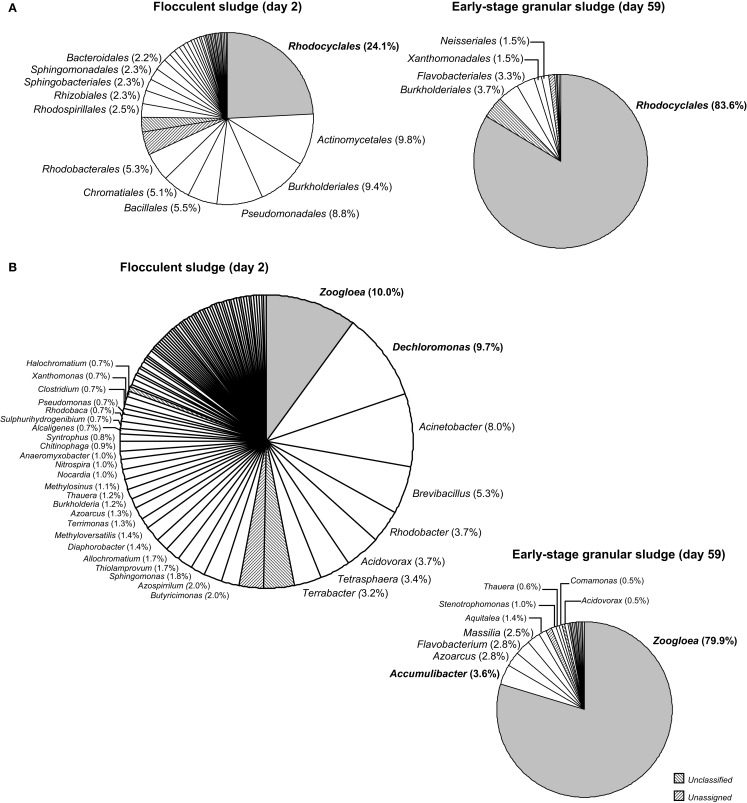
**Differences in the bacteriome composition of the flocculent sludge present in R6 before wash-out, and of the early-stage granular sludge present at day 59, at the order level (A) and at the genus level (B)**.

*^1^Relative abundances of bacterial orders obtained after mapping in MG-RAST (Meyer et al., 2008). Full bacteriome phylogenetic tree and sector graph representations are available in Figures A1, A2 in Appendix*.

At the level of the nitrifiers, the pyrosequencing analysis enabled detection of ammonium- (AOB) and nitrite-oxidizing bacteria (NOB). AOB were only detected at relative abundances below 0.5% in the flocculent sludge, namely *Nitrosococcus* (0.24%), *Nitrosomonas* (0.12%), and *Nitrosovibrio* spp. (0.06%), and represented a biomass concentration of 0.012 g_VSS_ ^−1^. The NOB-related *Nitrospira* spp. were detected in higher abundance (1.02%) than *Nitrobacter* spp. (0.06%). The two genera together accounted for a biomass concentration of 0.032 g_VSS_ L^−1^. The AOB and NOB present in the flocculent sludge were not detected in the early-stage granules. Only the AOB *Nitrosospira* spp. were detected at 0.03%, and accounted for 0.002 g_VSS_ L^−1^ on this particular day.

## Discussion

### Fluffy and dense fast-settling granules harbored different predominant phylotypes

Unfavorable filamentous bulking occurring during early-stage granulation was related to the application of an insufficient SAV (1.8 cm s^−1^) in the case of an inoculum taken from OMR-WWTP, or when operation was conducted at higher mesophilic temperature (30°C). The bacterial community of slow-settling fluffy granules was dominated by filamentous *Sphaerotilus* and *Leptothrix* bacterial genera. These organisms are known to cause severe filamentous bulking in conventional WWTPs (Richard et al., 1985). During the formation of compact flocs and granular biofilms, the proliferation of filamentous organisms toward the outside of microbial aggregates is enhanced by substrate gradients generated by diffusion limitations across the biofilm matrices (Martins et al., 2004; Liu and Liu, 2006). The ecology data showed that filamentous bulking can also occur with acetate as carbon source, and not only with carbohydrates that have been proposed as main bulking vectors (Liu and Liu, 2006).

The application of a more intensive SAV (4.0 cm s^−1^) was successful for the recovery of smooth and dense fast-settling granules. In the study of McSwain et al. (2004), filamentous overgrowth was counteracted by high shear forces. In analogy to chlorine addition in conventional WWTPs, high shear forces helped to break the superficial filamentous structures. Specific remedial actions that suppress the cause of filamentous proliferation are however preferred for sustainable reactor operation (van Loosdrecht et al., 2008). The inoculation sludge taken from the BNR-WWTP was beneficial for the production of compact granules at 20°C with a low SAV. At full-scale level, this corresponds to definite energetic advantages. With the BNR-sludge, fluffy granules were only observed at 30°C. The growth kinetics of filamentous bacteria are enhanced at such temperature (Richard et al., 1985). In BNR-WWTPs, the successive anaerobic, anoxic, and aerobic zones are clearly separated. The readily biodegradable substrates are fully removed by PAO under anaerobic conditions, and are not available in the aerobic zone for fast-growing heterotrophs such as filamentous bacteria (van Loosdrecht et al., 2008). Thus, BNR-sludge exhibits a lower filamentous bulking potential than OMR-sludge, and can be advantageous for the granulation process. Ensuring full anaerobic acetate uptake in AGS-SBRs might also favorably suppress filamentous overgrowth.

Dense fast-settling early-stage aerobic granules were dominated by *Zoogloea* relatives. In contrast to fluffy and dense anaerobic granules that have been both dominated by *Methanosaeta* spp. (Grotenhuis et al., 1992; Hulshoff Pol et al., 2004), fluffy and dense aerobic granules were composed of different predominant phylotypes. *Zoogloea* spp. have also previously been detected in other granulation studies involving wash-out conditions (Etterer, 2006; Li et al., 2008; Ebrahimi et al., 2010; Gonzalez-Gil and Holliger, 2011).

### The possible role of *Rhodocyclales*-related organisms in granulation

The T-RFLP and metagenomics analyses revealed that *Rhodocyclales*-affiliated *Zoogloea*, *Dechloromonas*, *Thauera*, and *Rhodocyclus* spp. were abundant in the communities of fast-settling early-stage granules. *Acinetobacter* spp. were present during the transition from flocs to granules with anaerobic feeding. The *Rhodocyclales*-affiliated organisms share some physiological properties in BNR-WWTPs (Hesselsoe et al., 2009). They produce EPS and store poly-β-hydroxyalcanoates (PHA) when high organic loads are present under aerobic conditions, hold an arsenal of surface adhesins, and form flocs and biofilms (Sich and Van Rijn, 1997; Allen et al., 2004; Dugan et al., 2006; Oshiki et al., 2008; Nielsen et al., 2010; Seviour et al., 2012). Acetate was abundantly present under aerobic conditions due to pulse feeding, or to incomplete anaerobic uptake. Feast-famine regimes and high shear stress also stimulate EPS production during granulation (Liu and Tay, 2002; Dulekgurgen et al., 2008; Seviour et al., 2010). In contrast to flocculent sludge settling that can suffer from *Zoogloea*-mediated viscous bulking (Norberg and Enfors, 1982; van Niekerk et al., 1987; Lajoie et al., 2000), AGS settling was not hampered by the proliferation of *Zoogloea* relatives. High shear stress and compaction forces generated by up-flow aeration (Zima et al., 2007) were likely to counteract viscous bulking. In addition, storage compounds such as PHA confer higher density and settling velocity to bacterial cells (Mas et al., 1985; Schuler et al., 2001). PHA storage was confirmed by confocal laser scanning microscopy analysis with Nile Red staining of cross-sectioned granules dominated by *Zoogloea* spp. (data not shown). Hence, the physiology of *Zoogloea*-like and other *Rhodocyclales*-affiliated organisms might be relevant for the cohesion of granular biofilms. However, microbial aggregation is probably not restricted to single organisms, and specific process conditions could select for other organisms with similar functions (Bossier and Verstraete, 1996; Beun et al., 1999; Wang et al., 2009).

### Wash-out conditions as drastic bacterial selection pressure during aerobic granulation

Even though inoculation with BNR-sludge and anaerobic feeding were combined, active PAO and nitrifiers were outcompeted by *Zoogloea* spp. during start-up. Two tentative explanations of this specific bacterial selection were formulated from the results of R3–R5 based on wash-out dynamics. Firstly, the wash-out dynamics resulted in an insufficient SRT that did not enable bacterial populations with lower growth rates such as PAO and nitrifiers to maintain themselves in the system. Secondly, during anaerobic feeding, the influent wastewater was not long enough in contact with the low residual biomass after wash-out. With a constant volumetric OLR and a fixed anaerobic plug-flow feeding phase, a large acetate fraction was present during aeration and selected for fast-growing *Zoogloea* spp. over PAO. The data collected with R6 were used to confirm these explanations, and are discussed hereafter.

*Tetrasphaera* spp. and other *Rhodocyclales*-affiliated organisms such as *Dechloromonas*, *Methyloversatilis* spp., and *Rhodocyclus* spp. to a lower extent, were able to compete with *Zoogloea* spp. for the carbon source when 2.45 g_VSS_ L^−1^ and 30 cm of settled flocculent biomass was initially present in the system. By considering a bed porosity of 0.5 and an influent flow-rate of 21 mL min^−1^, each volume fraction of the influent wastewater was in contact with the settled biomass during 15 min on average. With this contact time, 50% of acetate was removed under anaerobic conditions with concomitant release of orthophosphate showing that PAO activity was still present. *Accumulibacter* was only present in low abundance in the flocculent sludge (0.1–0.5%). However, additional organisms could have contributed to the detected PAO activity. *Tetrasphaera* spp. have been described as putative PAO in full-scale BNR-WWTP, but their underlying dephosphatating metabolism has not yet been deciphered (Nielsen et al., 2012). *Dechloromonas* spp. have been described as an accompanying guild of *Accumulibacter*, and have been proposed as putative PAO as well (Kong et al., 2007; Oehmen et al., 2010). The genus *Methyloversatilis* that affiliates to *Rhodocyclales* has only recently been discovered, and has been shown to metabolize nitrogen (Kalyuzhnaya et al., 2006; Baytshtok et al., 2008; Kittichotirat et al., 2011). However, more research is required on its metabolism under alternating anaerobic-aerobic conditions.

The combination of a low settling time (3 min) and a low HRT (6 h) resulted in intensive biomass wash-out. The SRT dropped to a value close to the HRT, and the reactor system was governed by the hydraulic properties. Aerobic heterotrophic organisms such as *Zoogloea*, *Dechloromonas*, *Acinetobacter*, and filamentous *Burkholderiales* affiliates that are related to maximum growth rates of 0.229–0.690 h^−1^ (Lau et al., 1984; van Niekerk et al., 1987; Logan et al., 2001; Kim and Pagilla, 2003) that are above 1/HRT, were able to proliferate over slower-growing PAO (0.042 h^−1^, Henze et al., 1999) and nitrifiers (0.017–0.046 h^−1^, Xavier et al., 2007). During reactor start-up, strong decreases in richness and diversity were observed. The apparent decrease rates were about 3 times higher before than after wash-out, indicating that the use of a synthetic wastewater with acetate as sole carbon source significantly contributed to the change in the bacterial community structure before wash-out. Gonzalez-Gil and Holliger (2011) have also reported that early-stage AGS cultivated with acetate or propionate as sole carbon and energy sources displayed half of the richness of the inoculation sludge. Winkler et al. (2011) have reported that, although distant, denaturing gradient gel electrophoresis profiles of bacterial communities of a conventional WWTP and of a pilot AGS reactor fed with the same urban wastewater exhibited similar richness and eveness.

After wash-out, only 0.3 g_VSS_ L^−1^ of biomass was remaining in the system and the biomass specific OLR increased by a factor of 13 from 51 to 685 mg_CODs_ cycle^−1^ g_CODx_^−1^. Granulation started with a biomass specific OLR above 2.7 kg_CODs_ d^−1^ kg_CODx_^−1^ equivalents, which is in agreement with the bottom value of 1.3 kg_CODs_ d^−1^ kg_CODx_^−1^ considered by Morgenroth et al. (1997) to enable sludge granulation. However, with a settled biomass height of only 1 cm, the contact time with the influent wastewater was extremely short (30 s). The fixed anaerobic plug-flow feeding phase thus resulted in the leakage of more than 90% of the acetate load into the aeration phase where it was available for fast-growing aerobic heterotrophs. This also explains why *Zoogloea* spp. outcompeted *Accumulibacter*, and why phosphorus was not removed. Deteriorated dephosphatation has also been correlated in flocculent sludge SBRs with *Zoogloea* proliferation over PAO caused by the concomitant presence of acetate as electron donor and oxygen or nitrate as terminal electron acceptors (Fang et al., 2002; Montoya et al., 2008). Proper anaerobic selector operation has been recommended to suppress this zoogloeal overgrowth (van Loosdrecht et al., 2008).

In conclusion, the detailed microbial ecology investigation involving T-RFLP, pyrosequencing, and PyroTRF-ID analyses conducted in this study in combination with a bioprocess engineering approach showed that slow-settling fluffy granules and dense fast-settling early-stage granules cultivated under wash-out dynamics were displaying distinct predominant phylotypes, namely filamentous *Burkholderiales* affiliates and *Zoogloea* relatives, respectively. Filamentous bulking could be remediated by the application of intensive up-flow aeration, or by the use of an inoculation sludge taken from a BNR-WWTP. A combination of insufficient SRT and of leakage of acetate into the aeration phase was the cause for the proliferation of *Zoogloea* spp. in dense fast-settling granules, and for the deterioration of BNR performances which has been commonly observed by different authors during granulation start-ups. It is however not certain that *Zoogloea*-like organisms are essential in granule formation. Additional research is needed to determine if they are required to stimulate early-stage granulation in BNR systems, or if granules can be cultivated without their involvement. Furthermore, optimal operation conditions should be elucidated for maintaining a balance between organisms with granulation propensity and nutrient removing organisms in order to form granules with BNR activities in short start-up periods.

## Conflict of Interest Statement

The authors declare that the research was conducted in the absence of any commercial or financial relationships that could be construed as a potential conflict of interest.

## Appendix

Composition of the cultivation media (Table A1). Cloning-sequencing databank constructed for OTUs detected in reactors R1–R5 (Table A2). Full bacteriome phylogenetic tree constructed with the two pyrosequencing datasets (Figure A1). Sector graph representation of the bacteriome composition of the two bacteriomes of flocculent sludge and granular sludge (Figure A2).

**Table A1:** **Composition of the cultivation media, adapted from de Kreuk et al. (2005) and Ebrahimi et al. (2010)**.

Compound	CAS no.	Molecular formula	Molecular weight (g mol^−1^)	Amount per 20 L of medium^1^	Concentration in medium (mmol L^−1^)
**Carbon source medium**^2^					
Sodium acetate	127-09-3	C_2_H_3_O_2_Na ·3H_2_O	136.09	170.11/212.64 g	^3^62.5/78.1
Magnesium sulfate	7487-88-9	MgSO_4_·7H_2_O	246.51	17.75 g	3.6
Potassium chloride	7447-40-7	KCl	74.55	7.16 g	4.8
**Nitrogen and phosphorus sources medium**^2^					
Ammonium chloride	12125-02-9	NH_4_Cl	53.49	37.87 g	35.4
Dipotassium hydrogen phosphate	7758-11-4	K_2_HPO_4_	174.18	14.62 g	4.2
Potassium dihydrogen phosphate	7778-77-0	KH_2_PO_4_	136.09	5.72 g	2.1
Trace element solution	–	–	–	100 mL	–

**Compound**	**CAS no**.	**Molecular formula**	**Molecular weight (g mol^−1^)**	**Amount per 5 L of stock solution^1^ (g)**	**Concentration in stock solution (mmol L^−1^)**

**Trace element solution**					
Disodium ethylenediaminetetraacetate	139-33-3	C_10_H_14_N_2_O_8_Na_2_·2H_2_O	372.25	637.0	342.2
Zinc sulfate	7733-02-0	ZnSO_4_·7H_2_O	287.59	22.0	15.3
Calcium chloride	10043-52-4	CaCl_2_·2H_2_O	147.02	81.8	111.3
Manganese chloride	7773-01-5	MnCl_2_·4H_2_O	197.92	50.6	51.1
Iron(II) sulfate	7720-78-7	FeSO_4_·7H_2_O	278.05	49.9	35.9
Ammonium heptamolybdate	12027-67-7	(NH_4_)_6_Mo_7_O_24_·4H_2_O	1’235.88	16.4	2.7
Copper(II) sulfate	7758-98-7	CuSO_4_·5H_2_O	249.71	15.7	12.6
Cobalt(II) chloride	7646-79-9	CoCl_2_·6H_2_O	237.96	16.1	13.5

*^1^The cultivation media and the trace element solution were prepared in 20 and 5 L of demineralized water*.

*^2^During the feeding phase of each SBR cycle, the synthetic influent wastewater was composed of 10% (v/v) of carbon source medium, of 10% (v/v) of nitrogen and phosphorus source medium, and of 80% (v/v) of tap water*.

*^3^The reactors R1–R5 and R6 were operated with 400 and 500 mg_COD_ L^−1^ of acetate in the influent wastewater, respectively. The chemical oxygen demand (COD) conversion factor of acetate is 64 g_COD_ mol_Ac_^−1^*.

**Table A2:** **Phylogenetic affiliation of predominant clones isolated from biomass samples of reactors R1–R5**.

T-RF^1^ (bp)	Reactor^2^	Affiliation^3^		Accession number^3^	Closest relative and original microbiota^4^	Sequence identity^5^ (%)
		Phylum → Family	Genus	
71	R3	O: *Rhodocyclales*	G: *Zoogloea*	AJ505853	*Zoogloea resiniphila* strain PIV-3C2w from denitrifying bacterial community	99
185	R4	C: *Gammaproteobacteria*		AM180057	Uncultured bacterium clone A1-41 from a N- and P-removing system	97
		O: *Rhizobiales*	G: *Ochrobactrum*	CP000759	*Ochrobactrum anthropi* ATCC 49188	100
193	R4, R5	O: *Rhodocyclales*	G: *Dechloromonas*	AF314420	Uncultured bacterium PHOS-HE23 from aerobic EBPR system	99
195	R1, R3	O: *Rhodocyclales*	G: *Zoogloea*	DQ413172	*Zoogloea* sp. EMB 357 from anaerobic-aerobic SBR	100
	R4, R5	O: *Rhodocyclales*	G: *Zoogloea*	AF527582	Uncultured bacterium clone LPB19 from EBPR system	99
		O: *Enterobacteriales*	G: *Escherichia*/*Shigella*	AY838362	Bacterium HPC775 from effluent treatment plant of chemical and dye industry	98
208	R1, R3	O: *Burkholderiales*	G: *Sphaerotilus*	AB087568	*Sphaerotilus* sp. L19 involved in filamentous sludge bulking process	100
		O: *Burkholderiales*	G: *Leptothrix*	FM175128	Uncultured *Leptothrix* sp. from tufa core sample	98
	R4, R5	O: *Burkholderiales*		AB087576	*Leptothrix* sp. L18 involved in filamentous sludge bulking process	98
213	R5	O: *Burkholderiales*	F: *Comamonadaceae*	AY491594	Uncultured bacterium clone oc53 from oligotrophic microbial fuel cells	99
214	R4, R5	O: *Rhodocyclales*	F: *Rhodocyclales*	EF565147	Uncultured bacterium clone UWMH_4 in *Accumulibacter* enrichment	99
215	R3	O: *Rhodocyclales*	G: *Dechloromonas*	EF632559	*Dechloromonas* sp. A34 from phosphate mining overburden	98
	R4, R5	O: *Rhodocyclales*	G: *Dechloromonas*	AY823964	Uncultured *Betaproteobacterium* clone DFAW-071 from acetate-fed denitrifying community	100
216	R5	O: *Nitrosomonadales*	G: *Nitrosomonas*	AJ621027	Ammonium-oxidizing *Nitrosomonas* sp. Is32	97
217	R3	O: *Rhodocyclales*	G: *Zoogloea*	DQ413157	*Zoogloea* sp. EMB 108 from anaerobic-aerobic SBR	98
	R1	O: *Rhodocyclales*	G: *Thauera*	AM084110	*Thauera* sp. R-28312 from denitrifying community	95
224	R4, R5	O: *Sphingomonadales*	G: *Novosphingobium*	AJ746094	*Novosphingobium* sp. MG37 from haemodialysis water and fluid	100
		O: *Rhodobacterales*	F: *Hyphomonadaceae*	AF502221	Uncultured bacterium clone HP1B26 from EBPR sludge	99
239	R4, R5	C: *Gammaproteobacteria*		AY651306	Uncultured bacterium clone Cont82 from EBPR ecosystem	99
250	R3	O: *Pseudomonadales*	G: *Acinetobacter*	GQ383923	*Acinetobacter* sp. XJ127 from sewage water	99
	R4, R5	O: *Pseudomonadales*	G: *Acinetobacter*	DQ205305	Kartchner Caverns bacterium HI-O4	100
		O: *Flavobacteriales*	G: *Chryseobacterium*	AF502204	Uncultured bacterium clone HP1B06 from EBPR sludge	99
289	R4, R5	O: *Sphingomonadales*	F: *Sphingomonadaceae*	U37341	*Sphingomonas paucimobilis* isolate EPA 505 from PAH-degrading soil consortia	100

*The initial cloning-sequencing database of Ebrahimi et al. (2010) was complemented in the present study*.

*^1^Size of target terminal-restriction fragments (T-RF) obtained with *Hae*III digestion and forming operational taxonomic units (OTU)*.

*^2^Reactor biomass system from which the clones were isolated. A number of 1–7 clones were sequenced per T-RF*.

*^3^Closest bacterial affiliations and GenBank accession numbers obtain after mapping in the Ribosomal Database Project (RDP; Cole et al., 2009)*. *Legend: P, phylum; C, class; O, order; F, family; G, genus*.

*^4^Description of closest relatives and original microbiota from which the reference clones were isolated obtained from GenBank (Benson et al., 2011)*.

*^5^Sequence identity scores obtained after mapping in RDP*.
